# Reconstitution of Formylglycine-generating Enzyme with Copper(II) for Aldehyde Tag Conversion

**DOI:** 10.1074/jbc.M115.652669

**Published:** 2015-04-30

**Authors:** Patrick G. Holder, Lesley C. Jones, Penelope M. Drake, Robyn M. Barfield, Stefanie Bañas, Gregory W. de Hart, Jeanne Baker, David Rabuka

**Affiliations:** From Catalent Pharma Solutions, Emeryville, California 94608

**Keywords:** catalysis, enzyme kinetics, metalloenzyme, monoclonal antibody, post-translational modification (PTM), aldehyde tag, antibody drug conjugation

## Abstract

To further our aim of synthesizing aldehyde-tagged proteins for research and biotechnology applications, we developed methods for recombinant production of aerobic formylglycine-generating enzyme (FGE) in good yield. We then optimized the FGE biocatalytic reaction conditions for conversion of cysteine to formylglycine in aldehyde tags on intact monoclonal antibodies. During the development of these conditions, we discovered that pretreating FGE with copper(II) is required for high turnover rates and yields. After further investigation, we confirmed that both aerobic prokaryotic (*Streptomyces coelicolor*) and eukaryotic (*Homo sapiens*) FGEs contain a copper cofactor. The complete kinetic parameters for both forms of FGE are described, along with a proposed mechanism for FGE catalysis that accounts for the copper-dependent activity.

## Teaser

**Background:** Aerobic formylglycine-generating enzyme (FGE) converts cysteine to formylglycine *in vivo*.

**Results:** Purified FGE requires preactivation with copper to convert cysteine to formylglycine *in vitro*.

**Conclusion:** FGE is a metalloenzyme. It is also a useful biocatalyst for the production of proteins that contain aldehyde tags.

**Significance:** Understanding FGE biochemistry informs research on sulfatases and enables expanded biotechnology applications of the aldehyde tag.

## Introduction

The aerobic class of formylglycine-generating enzyme (FGE)^2^ (sulfatase-modifying factor 1, SUMF1, E.C. 1.8.99.-) is an oxidase that catalyzes the conversion of cysteine to formylglycine (fGly) (1). FGE recognizes a five-amino acid consensus sequence (C*X*P*X*R), and converts Cys within that sequence to fGly (2). In nature, FGE is required for the endogenous activation of sulfatases (3). Sulfatases contain an active site fGly that participates in sulfate ester hydrolysis (4). Inhibitory mutations to FGE in humans result in a debilitating condition known as multiple sulfatase deficiency (5). FGE is expressed broadly across cell types and organisms, and the FGE consensus sequence is highly conserved across all taxonomic kingdoms, including eukaryotes and prokaryotes. Although the sequence determinants for fGly formation are understood (2), the mechanism through which Cys is converted to fGly remains unclear (6–9).

Beyond its role in the active site of sulfatases, the aldehyde functional group in fGly is a particularly useful site for protein bioconjugation (10–14). It is an electrophilic carbonyl, which is not found among the 20 proteogenic amino acids, and aldehydes rarely occur as post-translational modifications; oxidized glycans are a notable exception (15). Accordingly, fGly stands out as an optimal choice for bioorthogonal conjugation. A variety of bioconjugation reactions are available to target carbonyls (13). It is possible to use hydrazines and alkoxyamines to form hydrazones and oximes, but these are susceptible to hydrolysis (14). Newer methods anchor payloads with stable chemistries that form C-C bonds, including Pictet-Spengler (16–18) and trapped-Knoevenagel (19) ligations.

**FIGURE 1.**
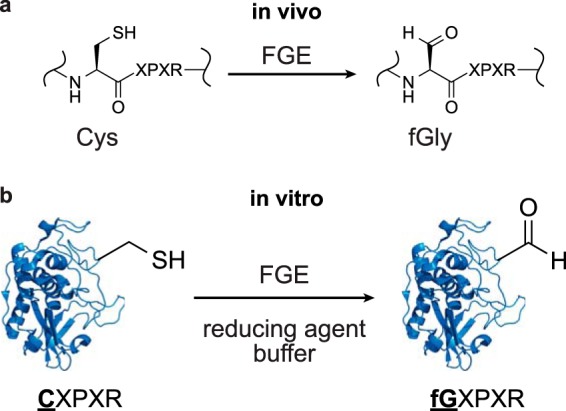
**FGE converts Cys to fGly *in vivo* and *in vitro*.** *a*, FGE recognizes the five-amino acid consensus sequence C*X*P*X*R. It catalyzes the oxidation of the Cys thiol to an aldehyde to generate fGly. In eukaryotes, conversion occurs in the ER. *b*, in this work, we demonstrate that FGE is also an efficient biocatalyst *in vitro*. Treatment of an aldehyde-tagged substrate protein with FGE and a reducing agent generates fGly on folded proteins in high yield.

To leverage these exquisitely selective reactions for synthetic purposes, fGly must be installed into the protein backbone. Fortunately, the method for its incorporation requires only genetic encoding of the consensus sequence (dubbed the aldehyde tag). During translation of the substrate protein in the ER, FGE converts Cys to fGly (Fig. 1*a*). In previous work, we described methods for the overexpression of FGE with substrate proteins modified with the aldehyde tag (11). This approach endows the resulting protein with fGly during production in cell culture.

In parallel work described here, we explored the value of performing conversion of Cys to fGly *in vitro* with purified recombinant FGE (Fig. 1*b*). This goal requires an efficient enzyme that is stable during the course of a biocatalytic reaction. It was not known whether FGE would meet these requirements, and so our first priority was to optimize FGE *in vitro* conversion efficiency and protein stability. Our initial experiments employed a 14-amino acid peptide substrate, with which we determined the optimal conditions for *in vitro* conversion. This model system also allowed us to explore the mechanism through which FGE converts Cys to fGly.

Researchers currently propose a mechanism for FGE that does not require an exogenous cofactor for oxygen activation or catalysis. In a variety of experiments to date, x-ray crystallography (8, 9, 20, 21) and biochemical investigations (7, 9, 22) have only revealed the need for the addition of calcium for structural support of protein folding. Mechanistically, it is suggested that Cys^336^ directly activates molecular oxygen to form a transiently oxidized form of Cys, which is thought to perform substrate oxidation (6, 7, 9). If true, this mechanism of catalysis would be unique in all of biology.

However, during the process of optimizing FGE reaction conditions, we discovered that both the *Streptomyces coelicolor* (*Sc*-FGE) and *Homo sapiens* (*Hs*-FGE) forms of FGE are many times more active when they contain a copper cofactor. This result is in clear contrast to the existing hypothesis, and yet copper-centered oxygen activation is a common mechanism in biology; many enzymes use copper to lower the high activation barrier of the spin-forbidden reaction with ground-state triplet O_2_ (23). From the results detailed here, we now propose that aerobic FGEs are oxygen-activating copper oxidases. Furthermore, using copper-loaded FGE, we demonstrate efficient *in vitro* conversion of Cys to fGly on an intact monoclonal antibody (mAb).

FGE is found across a wide range of organisms, including prokaryotes and eukaryotes. Revealing that copper is a required cofactor for FGE catalysis explains how the enzyme is active in both the reducing cytosol (prokaryotes) and the oxidizing endoplasmic reticulum (eukaryotes); turnover requires copper and oxygen, and not an active site disulfide. In addition, by defining a biocatalytic protocol for *in vitro* conversion of Cys to fGly, we enable a straightforward, reliable way to install a site-specific, bioorthogonal functional group on any folded protein. These abilities mutually reinforce the unique utility of FGE for aldehyde production *in vivo* and *in vitro*.

## Experimental Procedures

### 

#### 

##### Estimation of Uncertainty

Measurements of uncertainty represent 95% confidence intervals calculated from the mean ± S.E. for a sample size (*n*) using the following equation,

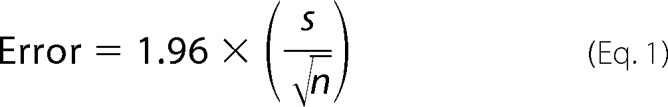
 where *s* is the measured standard deviation for *n* samples. Data without error bars represent individual experiments. The sample size *n* for data displaying error bars are indicated in each figure caption. Errors reported for the enzyme kinetic parameters represent the estimate of error calculated from the minimized sum of squares found during nonlinear regression of activity data to the Michaelis-Menten equation,

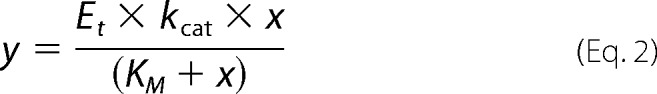
 where *E_t_* is the total enzyme in solution, and *k*_cat_ and *K_m_* are the standard enzymatic parameters specified by the STRENDA commission (24). Units of enzyme specific activity are the katal, where 1 katal = 1 mol s^−1^. Kinetic parameters were determined from nonlinear regression of [substrate] *versus* initial velocity using Prism® 6.0 (GraphPad).

##### RP-HPLC

Reversed-phase high performance liquid chromatography was performed on an 1100/1200 series instrument (Agilent Technologies). Chromatography was achieved on an Aeris^TM^ core-shell 250 × 2.1-mm XB-C18 Widepore column (Phenomenex, Inc.) and area under the curve was calculated with Chemstation (Agilent).

##### LC-MS/MS

Mass Spectrometry data were collected on a 4000 QTRAP® mass spectrometer (AB Sciex) with an 1100 series HPLC (Agilent). Chromatography was performed on a Jupiter^TM^ 150 × 1.0-mm C18 column (Phenomenex) enclosed in a butterfly column heater set to 65 °C with a PST-CHC controller (Phoenix S&T). Calculation of LC-MRM (multiple reaction monitoring)/MS transition masses and integration of the resulting data were performed with Skyline 2.6 (25).

##### FPLC and Gel Filtration

Fast performance liquid chromatography was performed on a GE Healthcare Äkta Protein Purification System. Nickel affinity chromatography was performed with a HisTrap Excel 5-ml column (GE Healthcare). Gel filtration was performed by hand with disposable Sephadex® G-25 columns (GE Healthcare).

##### ICP-MS

Inductively coupled plasmon mass spectrometry (ICP-MS) was performed by the Catalent Center for Excellence in Analytical Services (Morrisville, NC). The ratio of copper and calcium were calculated as a mole ratio based upon protein concentrations measured from 280 nm absorption intensity of enzyme stock solutions.

##### Reagents

Water used was deionized (18 mΩ) with a MilliQ® Integral 5 system (Merck KGaA). Other commercially available chemicals were reagent grade or higher. Peptide synthesis was performed by New England Peptide, Inc. on solid phase and purified to ≥95%.

##### Recombinant Expression and Purification of Prokaryotic FGE from Escherichia coli

Recombinant expression and purification of N terminally His_6_-tagged FGE from *S. coelicolor* (*Sc-*FGE, UniProt Q9F3C7) was carried out by modifying a previous method (9). Two significant changes were employed. First, residual DNA was removed from lysed cells using 1% (w/v) streptomycin sulfate for 15 min at 4 °C; then, the lysate was clarified by centrifugation at 25,000 relative centrifugal force for 25 min. Second, the enzyme purification and storage buffer included triethanolamine in place of Tris. The primary amine of Tris buffer was found to react with the aldehyde product of FGE catalysis. Additionally, it is worth noting that high ionic strength buffers (*e.g.* phosphate and sulfonate-containing Good's buffers) caused unactivated *Sc*-FGE to precipitate, and should be avoided.

After DNA precipitation, the cell lysate was loaded onto Ni-NTA Superflow (Qiagen) by gravity flow. The resin was washed with 10 column volumes of wash buffer (25 mm triethanolamine (TEAM), 250 mm NaCl, 5 mm calcium acetate, 10 mm imidazole, pH 8). The enzyme was then removed from the column with elution buffer (25 mm TEAM, 5 mm calcium acetate, 300 mm imidazole, pH 8). Fractions containing protein as determined by a protein assay (Bio-Rad) were pooled and loaded onto a Sephadex® G-25 column equilibrated with storage buffer (25 mm TEAM, 8% (v/v) glycerol, pH 7.4). Then, the protein was eluted with storage buffer, concentrated with a 10-kDa Amicon® ultrafiltration membrane (Merck KGaA), and flash frozen in liquid nitrogen. The typical yield of isolated enzyme was 50–75 mg/liter of media. Purified enzyme was characterized by SDS-PAGE electrophoresis, reversed-phase HPLC, LC/MS of the intact protein, tryptic digestion followed by LC-MS/MS, absorption spectroscopy, and specific activity (as described below).

##### Production of Recombinant Baculovirus Encoding for the Core of Human FGE

DNA encoding the catalytic “core” of *H. sapiens* FGE followed by a C-terminal His_6_ tag for purification (*Hs*-cFGE) was amplified from *Hs*-FGE (Uniprot Q8NBK3) by PCR using the following primers: forward, 5′-GAGGCTAACGCTCCGGGCCC-3′; reverse, 5′-GTCCATAGTGGGCAGGCGGTC-3′. Preparation of the *Hs-*cFGE-encoding baculovirus was performed using the Bac-to-Bac baculovirus expression system (Invitrogen).

##### Expression and Purification of Hs-cFGE from Hi5 Cells

*Hs*-cFGE baculovirus was transfected into Hi5 cells (Invitrogen) at an multiplicity of infection of 0.1. After 72 h in culture, the conditioned media was clarified by centrifugation and filtration (0.45 μm), and loaded by FPLC onto HisTrap® Excel resin (GE Healthcare) at 5 ml/min. The resin was washed with 10 column volumes of wash buffer (25 mm TEAM, 250 mm NaCl, 5 mm calcium acetate, 10 mm imidazole, pH 8). The enzyme was then removed from the column with elution buffer (25 mm TEAM, 5 mm calcium acetate, 300 mm imidazole, pH 8). Fractions containing protein (as determined by UV detection of the FPLC elution) were pooled and loaded onto a Sephadex® G-25 column (GE Healthcare) equilibrated with storage buffer (25 mm TEAM, 8% (v/v) glycerol, pH 7.4). Then, the protein was eluted with storage buffer, concentrated with a 10-kDa Amicon® ultrafiltration membrane, and flash frozen in liquid nitrogen. The typical yield of isolated enzyme was 10–20 mg/liter of media. Purified enzyme was characterized by SDS-PAGE electrophoresis, reversed-phase HPLC, LC/MS of the intact protein, tryptic digestion followed by LC-MS/MS, absorption spectroscopy, and specific activity (as described below).

##### Assay of FGE Catalytic Activity

The specific activity of FGE was measured using a discontinuous enzymatic activity assay (24). The substrate for FGE was an unmodified 14-amino acid peptide, ALCTPSRGSLFTGR, was derived from the wild-type sequence contained in a bacterial sulfatase (26). The rate of reaction was determined by integrating the peak areas at 215 nm of the substrate cysteine (C_sub_) and product (fGly) peptides on reversed-phase HPLC. The identities of product (and side product) peaks were determined by RP-HPLC MS/MS (see below). Each aqueous reaction solution (120 μl) contained C_sub_ (100 μm), FGE (0.1–1 μm), DTT (1 mm), and buffer (25 mm TEAM, pH 9). A single time course consisted of 5 data points collected at evenly spaced intervals. The reaction was initiated upon addition of FGE stock solution and vortexing for 3 s. The reaction vial was vortexed at 1500 rpm and 25 °C in a thermomixer R (Eppendorf). Time points were hand quenched by adding 20 μl of reaction mixture to 2 μl of 1 m HCl. After completion of the reaction, each time point was analyzed by RP-HPLC (see below).

##### Identification of FGE Peptide Intermediates by LC-MS/MS and RP-HPLC

To identify the intermediates and products of *in vitro* conversion by FGE on C_sub_, reaction mixtures from the FGE activity assay were quenched and analyzed by LC/MS. The reaction mixture consisted of 0.5 mm peptide, 0.5 μm FGE, 5 mm βME, and 25 mm TEAM, pH 9, in a total volume of 200 μl. Time points were prepared by adding 20 μl of reaction mixture to 2 μl of 1 m HCl. The gradient was 5–25% MeCN in water with 0.1% formic acid over 10 min. The relative intensities and retention times of the four peptide peaks detected at 215 nm on RP-HPLC were replicated in the LC-MS/MS data, allowing assignment of each peak by mass.

##### Quantification of FGE Substrate and Product Peptides by HPLC

Substrate, product, and side products of the FGE substrate peptide ALCTPSRGSLFTGR were separated on RP-HPLC over 7 min with isocratic 18% MeCN in water containing 0.1% TFA. The integrated areas of the fGly (2.1 min), C_sub_ (3.3 min), the C_sub_/βME disulfide (C_sub_-βME, 3.6 min), and substrate/substrate disulfide (C_sub_-C_sub_, 6.1 min) forms of the substrate were used to calculate the total area and each fraction thereof.

##### Thiol and Disulfide Mapping of FGE

To determine the oxidation state of thiol residues on either *Sc*-FGE or *Hs*-FGE, a procedure was developed for mapping thiols and disulfides by LC-MRM/MS using sequential labeling, digestion, and orthogonal labeling. Step 1 was initial labeling and tryptic digestion: the reaction mixture was composed of FGE (7.5 μg), NH_4_HCO_3_ (50 mm), iodoacetamide (20 mm), and trypsin (0.75 μg) in a total volume of 30 μl. The tube containing the sample was incubated at 37 °C for 16 h. Step 2 was quenching: water (6 μl) and DTT (4.8 μl of 0.5 m, 50 mm final concentration) were added to the reaction mixture from step 1. The tube containing the sample was incubated at 37 °C and 1500 rpm for 1 h. Step 3 was a second labeling, *N*-ethylmaleimide (7.2 μl of 0.5 m, 150 mm final concentration) was added to the reaction mixture from step 2. The tube containing the sample was then incubated at 37 °C and 1500 rpm for 1 h. After step 3, the reaction mixture was quenched with HCl (4.8 μl of 1 m, 100 mm final concentration). Digested peptides were separated with a gradient of 0–50% MeCN in water with 0.1% formic acid over 15 min. Cysteine-containing peptides were detected by MRM/MS monitoring iodoacetamide, *N*-ethylmaleimide, and oxidative modifications of Cys residues.

##### Activation of FGE with Copper(II) Sulfate

Copper sulfate (50 μm, 5 molar eq) was added to a solution of *Sc*-FGE (10 μm) in buffered aqueous solution (25 mm TEAM, pH 7.4, 50 mm NaCl). The mixture was vortexed at 1500 rpm for 1 h at 25 °C. The protein was purified from the reaction mixture by buffer exchange into 25 mm TEAM, pH 7.4, 50 mm NaCl using Sephadex® G-25 resin. Then, the specific activity of FGE was measured using the activity assay described above. Proteins containing a His_6_ tag were prone to aggregate during activation. Addition of 10 molar eq of EDTA after activation reversed this aggregation without removing copper from the enzyme. Alternatively, TEV protease was used to remove the tag before activation, which resulted in homogenous reaction solutions.

##### Generation of FGE Apoenzyme

EDTA (15 mm) and tris(carboxyethyl)phosphine (TCEP, 15 mm) were added to a solution of *Sc*-FGE holoenzyme (5 μm) in buffered aqueous solution (25 mm TEAM, pH 7.4, 50 mm NaCl). The mixture was vortexed at 750 rpm for 1 h at 37 °C. The protein was purified from the reaction mixture by buffer exchange into 25 mm TEAM, pH 7.4, 50 mm NaCl using Sephadex® G-25 resin. Then, the specific activity of FGE was measured using the activity assay described above.

##### Quantification of fGly Content in an Intact mAb

GPEx® technology (27) was used to generate bulk stable pools of antibody-expressing CHO cells, and monoclonal antibodies were purified from the conditioned medium using Protein A chromatography on MabSelect SuRe® resin (GE Healthcare). DTT (20 mm) was added to a solution of mAb (20 μg) in buffered aqueous solution (25 mm NH_4_HCO_3_) to a final volume of 20 μl. The tube containing the sample was incubated at 37 °C for 15 min at 1500 rpm. Then, HCl (50 mm) was added to the solution and the tube was vortexed until mixed. Next, ammonium bicarbonate (100 mm) was added to the solution and the tube was vortexed until mixed. Trypsin (1 μg) and iodoacetamide (50 mm) were added, and the tube was incubated at 37 °C and 1500 rpm for 60 min in the dark. Finally, sodium citrate (150 mm, pH 5.5) and methoxylamine (100 mm) were added to the solution, and the tube was incubated at 37 °C for 16 h. The processed sample was then analyzed by LC-MRM/MS. The MRM transitions included modifications of the Cys-containing peptide including: cysteine, cysteine modified with iodoacetamide, formylglycine, formylglycine hydrate (including loss of water in the collision cell), and formylglycine methyl oxime. The abundance of each species was defined by its 5 most intense precursor/product ion pairs, which were integrated to give the total signal and relative fractions of each component. In the optimized digestion and capping procedure described above, we did not detect unreacted Cys, fGly, or fGly-hydrate above background, observing only carboxyacetamidomethyl-Cys (CAM) and the methyl oxime of fGly (MeOx).

##### In Vitro Conversion of Intact mAb with FGE

*Hs*-cFGE was added to a solution of IgG containing the aldehyde tag (where the C-terminal Lys was substituted with SLCTPSRGS) in 25 mm TEAM, pH 9.0, with 50 mm NaCl and 1 mm βME. The enzyme was added at 10 mol % relative to the concentration of C_sub_ within the aldehyde tag of the antibody heavy chain. The solution was vortexed at 1000 rpm for 16 h at 18 °C, and then the solution was quenched with 0.25 volume eq of 0.5 m sodium citrate, pH 5.5 (to a final concentration 100 mm citrate), to decrease the pH of the solution to ∼7 for binding to MabSelect SuRe® protein A resin. The IgG was purified using standard methods. A typical reaction yielded a final fGly content in the antibody of 95–100%, corresponding to a reaction conversion yield of 90–100%. To date, the reaction has been performed across a range of scales from 20 μg to 0.6 g.

##### Competitive ELISA

Competitive ELISA plates (Maxisorp) were coated with 1 μg/ml of His-tagged antigen (Sino Biological) at 1 μg/ml in PBS for 16 h at 4 °C. The plate was then washed four times with PBS, 0.1% Tween 20 (PBS-T), and then blocked with 200 μl of ELISA blocker (Thermo). In a second plate, an 11-point standard curve was prepared from serial 3-fold dilutions (50 μl + 100 μl) of a stock solution of the competitor antibody at 20 μg/ml into ELISA blocker. Next, biotin-tagged antigen (100 μl of 10 ng/ml) was added to all of the wells. Finally, the mixture of competitor and biotin-tagged antigen was transferred to the coated ELISA plate (100 μl/well), which was incubated at room temperature for 1–2 h with shaking. The final concentration of biotin-tagged antigen was 5 ng/ml and the highest concentration of the competitor was 10 μg/ml. The plate was washed four times with PBS-T; then, 100 μl of a 1:10,000 dilution (into blocking buffer) of streptavidin-HRP (Thermo) was added and the plate was incubated at room temperature with shaking for 1–2 h. Next, the plate was washed four times with PBS-T, and developed with 1-Step Ultra TMB (100 μl/well). The reaction was quenched with 50 μl of 2 m sulfuric acid, and absorbance was measured at 450 nm. EC_50_ values were determined by nonlinear regression.

## Results

### 

#### 

##### Sc-FGE and Hs-cFGE Have Modest Catalytic Activity as Isolated from Cell Culture

To develop a highly efficient, soluble form of FGE for use as a reagent in a biocatalytic reaction, we first developed reliable methods for its recombinant expression. FGEs from two species, *S. coelicolor* and *H. sapiens*, were selected for study because they had been previously characterized to some extent (7–9), and because they represented both prokaryotic and eukaryotic forms of the enzyme. In eukaryotes, FGE resides in the ER, an oxidizing environment; in prokaryotes, it resides in the cytosol, a reducing environment. These two forms of the enzyme are highly homologous in primary sequence and structure. We hoped to determine how highly similar FGEs are active in both of these different redox environments.

*Sc*-FGE was prepared in a similar manner to previous reports, by transformation of *E. coli* and purification from clarified cell lysates. *Hs*-cFGE was prepared in high purity and good yield by viral transduction of insect cells. We elected to produce the human form in insect cells because it expressed poorly in *E. coli*, perhaps because it bears both structural disulfides and an *N*-linked glycan.

Initial results indicated that FGEs from both species were prone to precipitation depending on buffer conditions. We eventually optimized the buffer to minimize this problem. However, with respect to the human enzyme, we also removed a noncatalytic portion of the enzyme that appeared to worsen precipitation (described in detail later). With soluble enzyme in hand, we proceeded to evaluate the activity of FGEs as produced from cell culture, and the reaction conditions required for catalysis.

To measure the specific activity of FGE, we sought to develop an assay that could continuously read out product formation during turnover. We tested a variety of detection methods hoping to find an assay to monitor the quantity of substrate(s) and/or product(s). Notably, we attempted secondary conjugation to fGly, but found that the rate of turnover far exceeded the rate of any carbonyl condensation reaction. In addition, fluorescence detection of H_2_S (the hypothesized sulfurous leaving group) was unsuccessful because none of the sensors attempted (28, 29) were selective for H_2_S in the presence of other thiol reducing agents, which are, by necessity, added to the FGE reaction mixture.

In the end, a discontinuous assay was the most reliable and informative. We adapted a previously reported method that used MALDI-MS to quantify the starting material and product of quenched reaction mixtures (5). In our assay, reversed-phase HPLC was used to separate the Cys and fGly forms of a peptide containing the FGE consensus sequence, which were detected by optical absorbance. The 14-amino acid peptide ALCTPSRGSLFTGR (derived from a bacterial sulfatase sequence) was used as a substrate for both the *Sc-* and *Hs-* forms of the enzyme.

**FIGURE 2.**
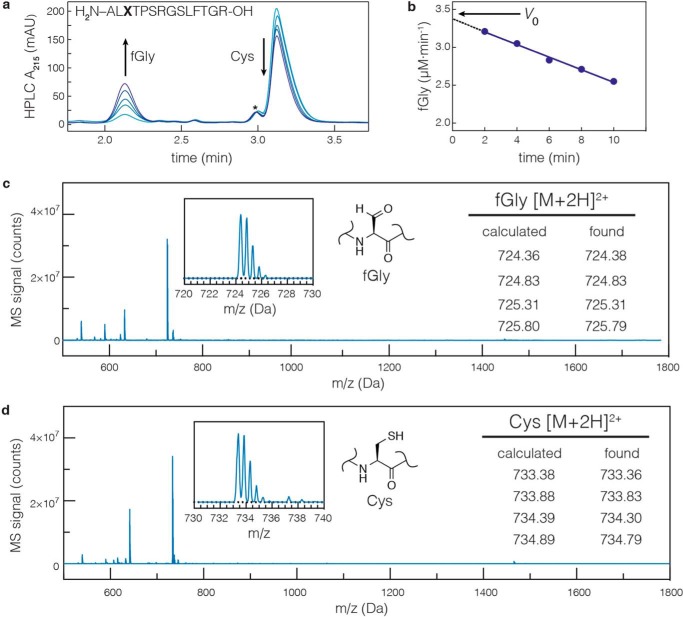
**The specific activities of FGEs are measured using a discontinuous activity assay.** *a*, a 14-amino acid peptide substrate was used to measure the kinetics of FGE catalysis. The Cys- and fGly-containing peptides were separated by RP-HPLC, and their quantities were determined by integrating the peak areas at 215 nm. The *asterisk* indicates an impurity formed during peptide synthesis. *b*, initial velocity of the reaction (*v*_0_) was determined by extrapolating a plot of the instantaneous reaction velocity (*p*/*t*) *versus* time (*t*) to the *y* axis (*t* = 0). The identities of the product (*c*, retention *t* = 2.1 min) and starting material (*d*, *t* = 3.3 min) were verified by LC-MS/MS.

Individual FGE reactions were initiated by rapidly mixing FGE into a premixed buffered solution of C_sub_ and reducing agent. It was assumed that all buffered solutions contained oxygen at ∼270 μm at 25 °C (based upon Henry's law). Time points were chemically quenched by hand with HCl. The integrated peak areas of substrate and product (Fig. 2*a*) were used to calculate the initial velocity (Fig. 2*b*) of FGE using the method described by Boeker (30, 31). The identities of both the Cys- and fGly-containing peptides were confirmed by LC-MS/MS (Fig. 2, *c* and *d*). As will be described below, the specific activity of FGE can vary dramatically as a function of the chemical state of its active site, and so enzyme concentrations in this assay spanned the range of 0.1–10 μm (0.1–10 mol %) enzyme.

**FIGURE 3.**
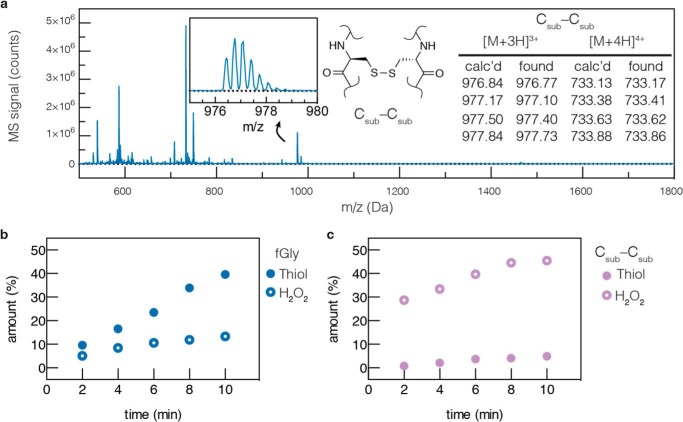
**Reaction inhibition upon treatment with H_2_O_2_.** *a*, the identity of the C_sub_-C_sub_ dimer was confirmed by LC-MS of a side product peak that formed during *in vitro* conversion with FGE. *b*, addition of hydrogen peroxide to FGE reaction mixtures inhibits product formation, and *c*, generates substrate dimer.

The conversion of Cys to fGly requires a 2 H^+^/2 *e*^−^ oxidation of the substrate Cys thiol as well as exchange of sulfur for oxygen. Current data support a mechanism in which molecular oxygen is the terminal electron acceptor (9). However, because O_2_ is a 4 H^+^/4 *e*^−^ acceptor, a reducing agent is required to provide a second equivalent of 2 H^+^ and 2 *e*^−^, completing the reduction from O_2_ to 2 mol of H_2_O. We hypothesized that one might be able to bypass the requirement for a reductant by adding a 2 H^+^/2 *e*^−^ oxidant directly to the *in vitro* reaction. Unfortunately, we found that addition of the oxidant H_2_O_2_ served only to dimerize the substrate by generating a disulfide (C_sub_-C_sub_, Fig. 3*a*), which halted the reaction (Fig. 3, *b* and c). We returned to using a mixture of reductant (DTT) and O_2_ for routine activity measurements. We now had the ability to produce active FGEs in good yield and high purity from both species, and we turned to developing methods that would enhance the activity of FGE. To do so, we investigated the mechanism by which FGE performs catalysis.

##### The Activity of FGE Depends Upon the Presence of a Copper Cofactor, and Not the Redox State of Active Site Cysteine Residues

**FIGURE 4.**
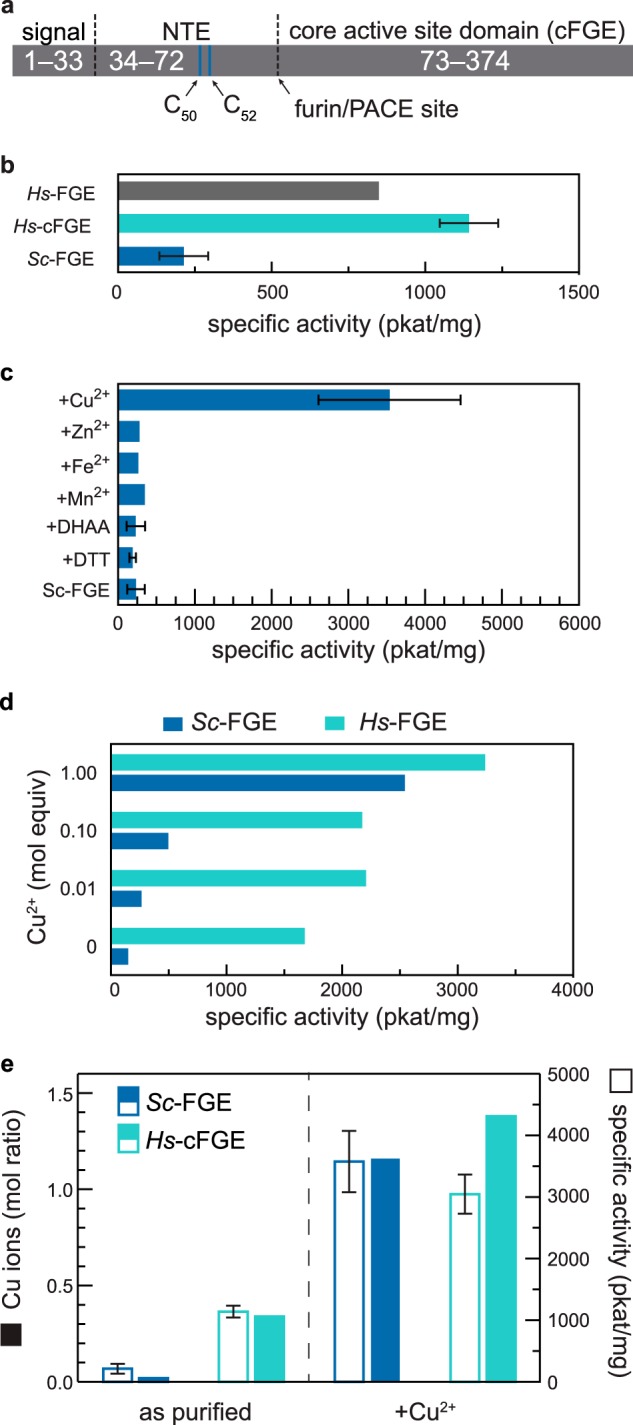
**The specific activity of FGE increases significantly upon treatment with stoichiometric amounts of copper(II).** *a*, wild-type *Hs*-FGE contains three primary domains: an ER-directing signal, an NTE that interacts with ERp44 for ER retention, and a catalytic core. *b*, specific activity of FGEs as purified from cell culture. Full-length *Hs*-FGE (*n* = 1) and the core lacking the NTE (*Hs*-cFGE, *n* = 10) have similar specific activities. The prokaryotic *Sc*-FGE as produced in *E. coli* was less active (*n* = 9) than *Hs-*cFGE. *c*, treatment of *Sc*-FGE with 2-electron oxidants or reductants (DHAA, *n* = 5; DTT, *n* = 2) does not change the activity of the enzyme. In contrast, treatment with an excess of CuSO_4_ followed by gel filtration to remove the Cu^2+^ significantly (*p* < 0.0001) increases FGE activity (*n* = 7). Other metal cofactors are unable to increase FGE activity (FeSO_4_, MnCl_2_, ZnCl_2_). *d,* FGE activation requires equimolar amounts of Cu^2+^. *e*, as purified, *Sc*-FGE contains ∼0.01 Cu^2+^/FGE, and *Hs*-cFGE contains 0.34 Cu^2+^/FGE. After reconstitution, *Sc*-FGE and *Hs*-FGE contain 1.1 and 1.4 Cu^2+^/FGE, respectively, and the amount of copper correlates with the specific activity of the enzyme.

*Hs*-FGE contains three primary domains: a signal peptide, an N-terminal extension (NTE), and a core that binds substrate and performs turnover (Fig. 4*a*). After transport to the ER and proteolytic removal of the signal peptide, the “full-length” wild-type enzyme (*Hs*-FGE) contains both the NTE and core. Previous reports provide evidence that the NTE is required for ER retention through disulfide bond formation between Cys^50^ and/or Cys^52^ with ERp44 (32). The border between the NTE and the core is a proteolytic cleavage site for proprotein convertases such as furin and PACE (33). When FGE encounters these enzymes in the secretory pathway, the NTE is removed and the truncated core can be secreted. We discovered that *in vitro*, the full-length FGE had a propensity to aggregate at high concentrations, which led us to prepare the truncated core (cFGE). We were unsure whether this approach would be successful because previous results showed that the NTE is required for FGE activity *in vitro* (33). In our hands, the specific activity of *Hs*-cFGE (1,143 pkatal mg^−1^, 33.3 kDa) was similar to *Hs*-FGE that contained the NTE (850 pkatal mg^−1^, 36.9 kDa). In addition, we found that *Sc*-FGE expressed in *E. coli* had a significantly lower specific activity (214 pkatal mg^−1^), relative to the human enzyme expressed in insect cells (Fig. 4*b*).

Our analysis of FGE catalysis then turned to testing the previously reported hypotheses of its mechanism. Foremost among these is that the enzyme requires the presence of an active site disulfide residue (8). Therefore, we prepared reduced and oxidized forms of FGE and measured their specific activities. Formation of the reduced active site Cys residues was accomplished by pretreatment of *Sc*-FGE with an excess (100 molar eq) of a strong reducing agent (DTT) for 1 h at 25 °C, after which the reagent was removed by gel filtration. Formation of the oxidized active site was accomplished by pretreatment of the enzyme either with an excess (100 molar eq) of l-dehydroascorbic acid or CuSO_4_ for 1 h at 25 °C, followed by removal of the reagents by gel filtration. l-Dehydroascorbic acid can form disulfides stoichiometrically (34), and Cu^2+^ can rapidly, and cleanly, catalyze disulfide formation with dissolved oxygen (35). After these treatments, the specific activities of each enzyme were measured using the standard kinetic assay. Only pretreatment with CuSO_4_ resulted in any significant change in activity (Fig. 4*c*). We were surprised by this result, because a previous report showed that addition of copper to FGE isolated from HT1080 cells *inhibited* activity (7).

Because pretreatment and removal of copper led to more active FGE, we next investigated the quantity of copper required to activate *Sc*-FGE. We hypothesized that copper might generate active enzyme by forming the active site disulfide, priming the enzyme for turnover, and thus might only be required in catalytic amounts. However, treatment of FGE with 0.01, 0.1, or 1 molar eq of CuSO_4_ demonstrated that generating a highly active enzyme requires stoichiometric amounts of copper (Fig. 4*d*). Any amount less than 1 molar eq resulted in proportionally lower activity, an observation that is inconsistent with the *catalytic* activation of active site thiols.

After optimization, we found that both *Sc-*FGE and *Hs-*cFGE could be efficiently activated with 5 molar eq of CuSO_4_ for 1 h at 25 °C followed by removal of excess copper. The specific activity of *Sc*-FGE increased by more than an order of magnitude, from 214 ± 34 pkatal mg^−1^ (*n* = 9) to 3579 ± 215 pkatal mg^−1^ (*n* = 7); *Hs*-cFGE specific activity increased by a factor of 3, from 1,143 ± 84 pkatal mg^−1^ (*n* = 10) to 3,050 ± 115 pkatal mg^−1^ (*n* = 5, Fig. 4*e*). Next, we tested whether FGE that was treated with copper contained the metal after purification. To answer this question, we measured the quantities of copper and calcium in both the unactivated and the activated FGE samples using inductively coupled plasmon mass spectrometry. The *Sc*-FGE produced in *E. coli* contained almost no copper above the level of the noise of the measurement. By contrast, *Hs*-cFGE, as isolated from insect cells, contained 0.35 mol of copper per mol of FGE. Most strikingly, both the copper-treated “activated” forms of FGE contained just above 1 mol of copper/FGE. Furthermore, the specific activity of each of these enzyme preparations correlated with the ratio of copper/FGE, suggesting that copper is an integral component of the active form of FGE (Fig. 4*e* and Table 1). All of the FGE preparations also contained ∼1–2 calcium atoms per FGE.

**TABLE 1:** **FGE preparations contain both copper and calcium as measured by inductively coupled plasmon mass spectrometry**

Preparation	[Protein]*^a^*	Calcium	Calcium	Calcium (ratio)*^b^*	Copper	Copper	Copper (ratio)
	μ*m*	μ*g/liter*	μ*m*		μ*g/liter*	μ*m*	
*Sc*-FGE	131.4	375	373.75	**2.84**	111	1.74	**0.01**
*Hs*-cFGE	204.3	98	97.4	**0.48**	4368	68.73	**0.34**
*Sc*-FGE + copper	64.5	55	54.99	**0.85**	4768	75.03	**1.16**
Hs-FGE + copper	69.7	106	105.59	**1.51**	6112	96.18	**1.38**

*^a^* Protein concentrations were measured using the absorption at 280 nm of stock protein solutions. Extinction coefficients for protein were calculated from the primary sequence of the enzyme using the analysis tools on the ExPASy bioinformatics server (51). For *Hs*-cFGE, ϵ = 85,745 m^−1^ cm^−1^ and molecular mass = 33,286 Da; for *Sc*-FGE, ϵ = 88,000 m^−1^ cm^−1^ and molecular mass = 37,432 Da.

*^b^* Ratio describes molar equivalents of metal *versus* FGE.

**FIGURE 5.**
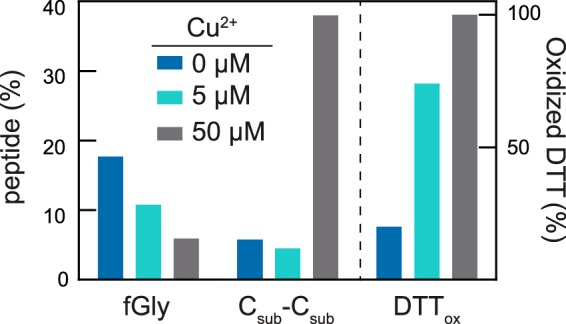
**Addition of Cu^2+^ to an FGE reaction mixture inhibits product formation.** Reactions were quenched after 10 min with 100 mm HCl, and the abundance of each species was determined by their integrated peak areas on HPLC. Product fGly peptide formation decreased with increasing amounts of Cu^2+^. This decrease in product formation is concomitant with oxidation of both DTT and substrate to produce oxidized DTT and C_sub_-C_sub_ homodimer.

We then considered whether it is possible to enhance the FGE reaction rate by adding copper directly to the reaction mixture. Instead, our data confirmed the previous report (7) that product formation was significantly inhibited by the inclusion of copper *in the reaction*. Specifically, we observed very rapid formation of the oxidized form of DTT as well as C_sub_-C_sub_ as a result of copper-catalyzed disulfide formation (Fig. 5).

**FIGURE 6.**
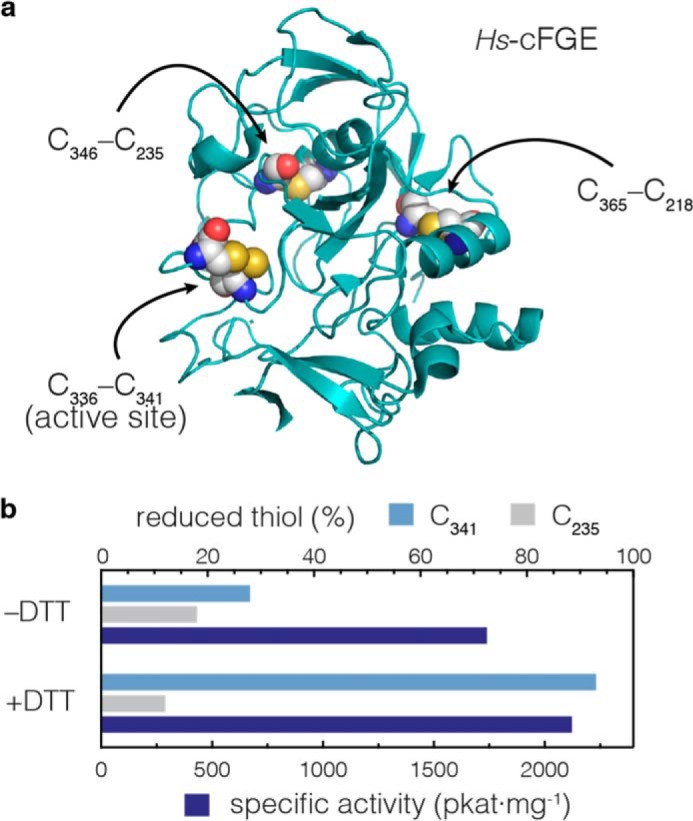
**Specific activity of *Hs*-cFGE does not correlate with the redox state of active site residue Cys^341^.** *a*, *Hs*-cFGE contains two structural disulfides and two active site cysteines, which can form a disulfide in the apoenzyme. *b*, the redox state of Cys^341^ can be measured by LC-MS/MS. After activation with Cu^2+^, the amount of Cys^341^ accessible to solution is ∼28%. Upon treatment with reducing agent (DTT) the amount of accessible Cys^341^ increases to 93%. However, the specific activity of the enzyme is not decreased. Treatment with DTT does not affect the Cys^235^-containing structural disulfide.

To confirm that catalytic activity in the activated form of FGE does not depend upon an active site disulfide, we tested whether addition of strong reducing agents to the *activated* enzyme would decrease the specific activity of the resulting material. If the integrity of the active site disulfide is indeed important for turnover, then strong reducing agents should have decreased FGE turnover rates. *Hs*-cFGE has the potential to form the active site disulfide (Cys^336^-Cys^341^) as well as two structural disulfides (Cys^346^-Cys^235^ and Cys^365^-Cys^218^) elsewhere in the protein (Fig. 6*a*). Treatment of *Hs*-cFGE with 20 mm DTT (or 20 mm TCEP, not shown) produced an FGE with unchanged specific activity (Fig. 6*b*). To confirm that the relevant Cys residues changed redox state as a result of this treatment, we developed an LC-MRM/MS assay to detect solution-accessible Cys residues in the active site. With this method, we successfully monitored both the Cys^341^- and Cys^235^-containing peptides. As expected, treatment with DTT increased the proportion of accessible active site Cys^341^ from 28 to 93%. Notably, Cys^235^, which participates in a buried structural disulfide, was inaccessible, and thus, in a disulfide independent of DTT treatment. Taken together, these data confirm that reductive treatment generated almost quantitatively reduced active site thiols, did not perturb other structural disulfides, but had no effect on the catalytic activity of FGE.

**FIGURE 7.**
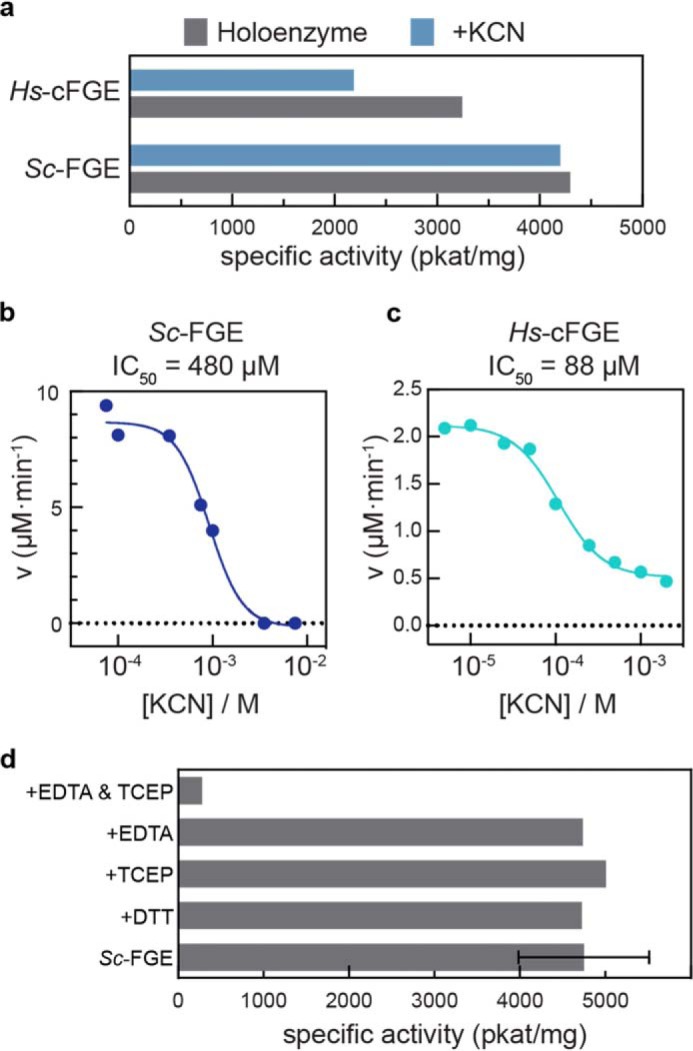
**FGE catalysis is inhibited by cyanide and EDTA in the presence of reductant.** *a*, pretreatment of activated FGEs with KCN (5 molar eq, 1 h, 25 °C) results in little change in specific activity; only *Hs*-cFGE activity decreases modestly. Both *Sc*-FGE (*b*) and *Hs*-cFGE (*c*) are inhibited during turnover in a concentration-dependent manner by CN^−^, which is a strong ligand for copper. *d*, pretreatment of activated *Sc*-FGE (*n* = 6) with reductants (DTT, TCEP) or a metal chelator (EDTA) do not change the specific activity of the enzyme. When the reductant and chelator are combined (15 mm each, 1 h, 37 °C), the FGE activity is nearly eliminated.

After observing that FGE contained ∼1 copper atom/enzyme, we made an effort to remove the metal from the protein to demonstrate its requirement for catalytic activity. Cyanide is known to be an inhibitor of copper-containing oxidases (36–38). Attempting to extract copper through pretreatment with 5 molar eq of KCN did decrease the activity of *Hs*-cFGE (but not *Sc*-FGE) by a modest amount, suggesting that it could gain access to the bound copper (Fig. 7*a*). We next tested whether FGE turnover could be inhibited competitively by cyanide. Indeed, we observed a concentration-dependent inhibition of activity on both *Sc*-FGE (IC_50_ = 480 μm, Fig. 7*b*) and *Hs*-cFGE (88 μm, Fig. 7*c*). In the case of copper amine oxidases, researchers had success generating apoenzyme by extracting copper with KCN from enzyme that was pre-reduced with sodium dithionite (39). In a similar manner, this approach was also successful for FGE. Activity decreased significantly upon treatment (1 h, 37 °C) with both a reductant and chelator (15 mm TCEP and EDTA), but not with each component individually (Fig. 7*d*).

**FIGURE 8.**
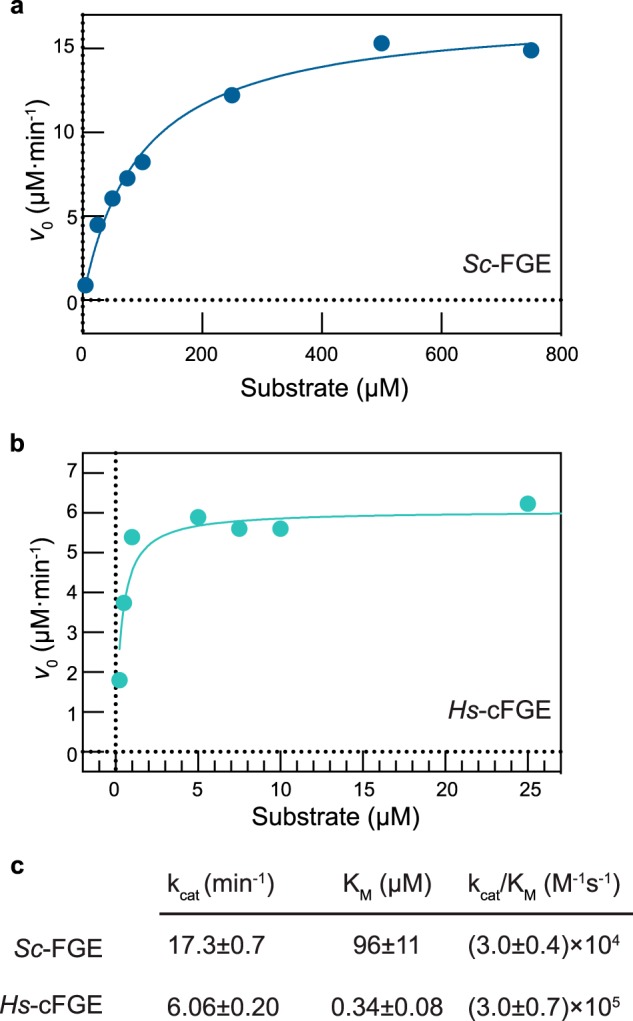
**Kinetic parameters of *Sc*-FGE and *Hs*-cFGE as determined by nonlinear regression.** *a*, Michaelis-Menten plot correlating substrate concentration with *v*_0_ for *Sc*-FGE, and *b*, for *Hs*-cFGE. The data were fit to the Michaelis-Menten equation by nonlinear regression to determine the kinetic parameters (*c*). *Errors* represent standard error of the regression.

Having discovered that Cu(II) generates the FGE holoenzyme, we next determined the standard enzymatic parameters of both *Sc*-FGE and *Hs*-cFGE (Fig. 8). Much to our surprise, despite their structural homology, the two species catalytic parameters were different. *Sc*-FGE did not interact with substrate very strongly (*K_m_* = 96 μm) and exhibited a relatively rapid *k*_cat_ (17.3 min^−1^), which together resulted in a modest enzyme efficiency with *k*_cat_/*K_m_* = 3.0 × 10^4^ m^−1^ s^−1^ (Fig. 8*a*). By comparison, *Hs*-cFGE turned over substrate with a slower catalytic rate constant (*k*_cat_ = 6.06 min^−1^) but demonstrated a strong interaction with the substrate peptide (*K_m_* = 0.34 μm). Taken together, the *Hs*-cFGE (*k*_cat_/*K_m_* = 3.0 × 10^5^ m^−1^ s^−1^) was ∼10-fold more efficient than its bacterial counterpart (Fig. 8*b*).

In total, our data demonstrate that the modestly active forms of FGE isolated from cell culture are a mixture of the apoenzyme, lacking the copper cofactor, and the holoenzyme, which is highly active and efficiently converts Cys to fGly on model peptide substrates. We then began developing robust conditions for using the FGE holoenzyme in biocatalytic reactions on folded protein substrates.

##### FGE Is an Efficient Biocatalyst for in Vitro Conversion of Cys to fGly on Folded Proteins

To use *in vitro* conversion for the production of aldehyde-tagged mAbs, we needed to modify the reaction conditions from those used for the kinetic assay. For most substrates, 5–10 molar eq of DTT was sufficient to achieve complete conversion. We found that turnover was also possible with TCEP, lipoic acid, and bis(2-mercaptoethyl)sulfone. However, the presence of even 1 molar eq of a strong reducing agent (*e.g.* DTT, TCEP, lipoic acid, or bis(2-mercaptoethyl)sulfone) can cleave disulfides that are present in a mAb substrate (40). To avoid substrate disulfide cleavage, we selected an alternate reducing agent for the reaction. We found that weaker reducing agents, *e.g.* βME or GSH, were tolerated by antibody substrates, and also enabled FGE catalysis. As it was most cost effective, we elected to move forward with βME for our antibody conversion reactions. However, we found that changing from a cyclizing (DTT) to a non-cyclizing reducing agent (βME) resulted in side products that had not previously been observed (discussed below). To reduce this side product formation and increase product yield, we further considered the role(s) of the reducing agent in product formation.

**FIGURE 9.**
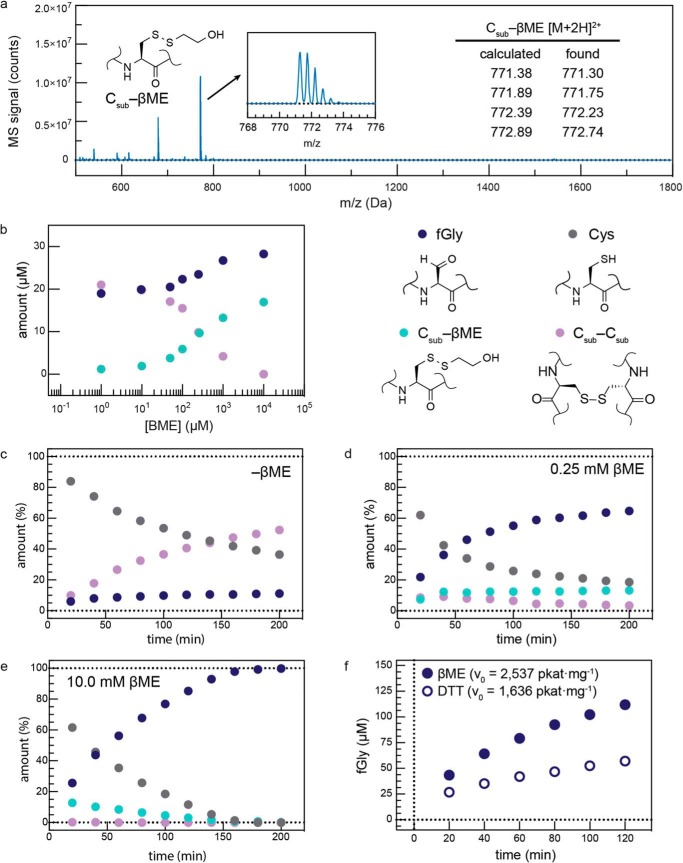
**FGE can catalyze substrate/reductant disulfide formation, which can be inhibitory to product yield, and confirms that substrate is bound in the active site as a disulfide.** *a*, LC/MS identification of C_sub_–βME. *b*, product formation in FGE reaction mixtures across a range of 0–10 mm βME. Without βME, fGly and C_sub_-C_sub_ form at comparable rates. As [βME] is increased, C_sub_-C_sub_ is replaced by C_sub_-βME, and product forms at a higher rate. Product formation over time catalyzed by FGE in the absence of reducing agent (*c*), with 2.5 mol eq of reducing agent (*d*), and with 100 mol eq of reducing agent (*e*). *f*, the initial velocity of FGE catalysis with βME is higher than with DTT (1 mm each reducing agent).

X-ray crystallography of FGE (what we now understand to be the apoenzyme) bound to a substrate peptide shows Cys^341^ and C_sub_ covalently linked by a disulfide in the active site (6). If the substrate is indeed anchored by this Cys^341^-C_sub_ disulfide during turnover, an exogenous thiol might be able to react with the enzyme-substrate complex (*E*·S) by thiol-disulfide exchange to release substrate in the form of a thiol-C_sub_ disulfide. Indeed, we observed two off-pathway products formed by FGE during turnover. First, when reducing agent was not added to the reaction, we observed C_sub_-C_sub_ dimer formation. Second, when we added a monothiol as the reductant (*e.g.* βME), we observed rapid production of the C_sub_-βME disulfide (Fig. 9*a*). In control reactions lacking enzyme, neither C_sub_-C_sub_ nor C_sub_-βME were generated (not shown).

To limit the formation of C_sub_-C_sub_ and C_sub_-βME, we monitored their formation as a function of reaction conditions. First, we varied the concentration of reducing agent in the reaction, and quenched the reactions when they were half-complete (Fig. 9*b*). At low [βME] *versus* [C_sub_], the C_sub_-C_sub_ predominated. Increasing [βME] resulted in a concurrent decrease in C_sub_-C_sub_, and an increase in C_sub_-βME. Importantly, increasing [βME] also resulted in a higher yield of fGly.

Second, we investigated the effect of [βME] as the reaction evolved over time. As mentioned above, in the absence of exogenous reducing agent product *did* form, but a significant proportion of C_sub_-C_sub_ was generated (Fig. 9*c*), suggesting that C_sub_ can act in place of the reducing agent during turnover, but at the expense of product yield. At low [βME] (0.25 mm, Fig. 9*d*), the C_sub_-C_sub_ initially grew in at a rate competitive with product formation, but as the reaction proceeded, it reached a maximum, and then decayed away slowly. At this [βME], the C_sub_-C_sub_ was consumed, but the C_sub_-βME reached a plateau of 13% of total peptide, where it remained, thereby limiting product yield. In a subsequent reaction with higher [βME] (10 mm, Fig. 9*e*), C_sub_-C_sub_ was almost undetectable. Moreover, C_sub_-βME rose to 10% of the total substrate, but it was consumed concomitant with product formation. In all cases, the consumption of both C_sub_-C_sub_ and C_sub_-βME correlated with a higher yield of product formation.

In contrast to βME, when we used DTT as the stoichiometric reductant neither the intermediate C_sub_-C_sub_ nor the C_sub_-DTT disulfides were observed. This is likely because DTT can cyclize to form an intramolecular disulfide and release substrate. However, we found that the rates of product formation in reaction using βME or DTT were not the same (Fig. 9*f*). Specifically, the rates of product formation were 5.07 or 3.27 min^−1^ in the presence of βME or DTT, respectively. Because reducing agent was present in large excess *versus* both the substrate and enzyme, it must be involved in limiting the rate of turnover.

If the reductants are acting by a 1-electron mechanism, their relative reaction rates should depend directly upon their reduction potentials: *E*_0_′(βME) = −207 mV and *E*_0_′(DTT) = −323 mV (41). If instead, the reductants are acting by thiol-disulfide exchange, their reaction rate should correlate with their thiol-disulfide exchange rate constants: *k*_βME_ = 1 and *k*_DTT_ = 6 × 10^5^, as measured *versus* glutathione disulfide (41). Both of these scenarios predict that reactions performed in the presence of DTT should have a faster rate than those performed in the presence of βME. By contrast, we observed that reactions performed with DTT were *slower* than those with βME, and so the role of the thiol in the reaction cannot be explained by the reaction rate of the thiol alone.

**FIGURE 10.**
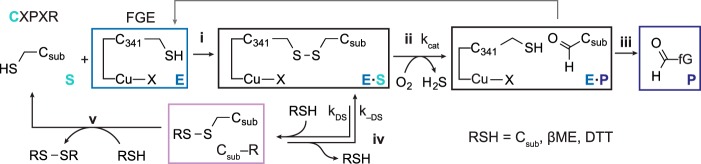
**The dual role of reducing agent in FGE catalyzed aldehyde tag conversion.** Formation of [*E*·S] results, (i) in a disulfide between *E* and S. Catalysis (ii), converts substrate to product, which is then released (iii). In the presence of thiol reducing agents, the rate of catalysis defined by *k*_cat_ is in competition with thiol-disulfide exchange (iv), with rate constant *k*_DS_, which dissociates the *E*·S complex and releases C_sub_-R. This species can then react with another equivalent of reducing agent (v), to regenerate free substrate. When the reagent added is a strong non-thiol reductant (*e.g.* TCEP) or a thiol that can cyclize (DTT), (iv) and (v) effectively collapse to a single step. When the reducing agent is a monothiol, the C_sub_-R may persist long enough to reform [*E*·S] with rate constant *k*_−DS_.

The covalent disulfide in *E*·S that was previously observed by crystallography is likely the source of the discrepancy just described (Fig. 10). From this state, *E*·S can either proceed to product with a rate constant of *k*_cat_, or it can react with an exogenous thiol to form a disulfide with a rate constant of *k*_DS_. For any given reducing agent, *k*_cat_ is likely the same based upon the mechanisms of analogous copper-dependent oxidases, in which active site redox chemistry is not rate-determining (42). However, *k*_DS_ will depend directly upon the identity of the reducing agent in solution and its reaction rate constant. Said another way, if *E*·S involves a disulfide, it should dissociate faster in the presence of a stronger reducing agent, in competition with product formation. If the substrate is released as a disulfide that does not decay quickly (as with βME-DS), it could react with the enzyme again to regenerate *E*·S. Therefore, stronger reductants should slow turnover by dissociating the *E*·S complex, as observed for DTT and βME.

The predominance of C_sub_-βME also confirms that the reducing agents are reacting with *E*·S by thiol/disulfide exchange: 1-electron reduction would regenerate free substrate thiol, and not a significant proportion of a substrate disulfide. Interestingly, this aspect of the cycle also explains the faster turnover rate observed at pH 9 relative to pH 7, thiol/disulfide exchange is faster under basic conditions because more of the nucleophilic thiolate is present.

**FIGURE 11.**
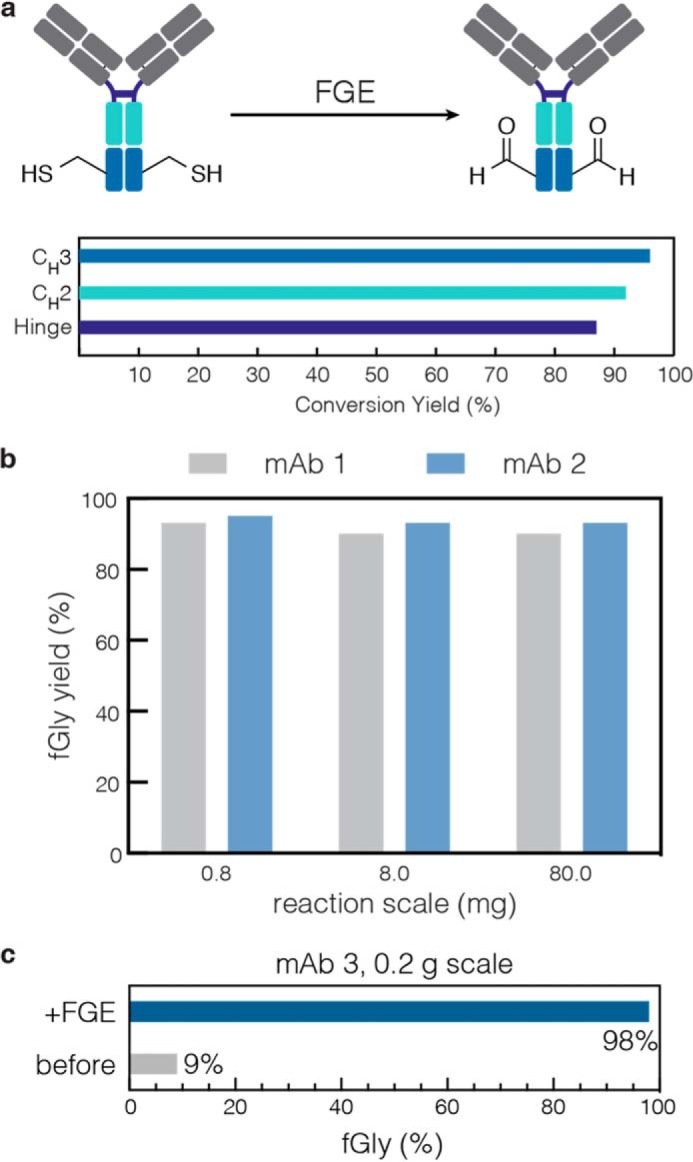
**FGE can be used to produce aldehyde-tagged mAbs in high yield.** *a*, the aldehyde tag was installed in three regions (hinge, C_H_2, and C_H_3) of the heavy chain of an IgG1. After reaction with FGE *in vitro*, the quantities of Cys and fGly were determined. Under optimized conditions (0.1 molar eq of FGE per C_sub_ + 1 mm βME), the yield of conversion from Cys to fGly was consistently 85–95%. *b*, the yield of biocatalytic fGly production is independent of the reaction scale, as measured in 0.8, 8.0, and 80.0 mg test cases on two different mAbs. *c*, representative example from a third antibody on a larger scale reaction. After conversion with FGE, the fGly content is 98.1%, corresponding to a conversion yield of 97.8%.

**FIGURE 12.**
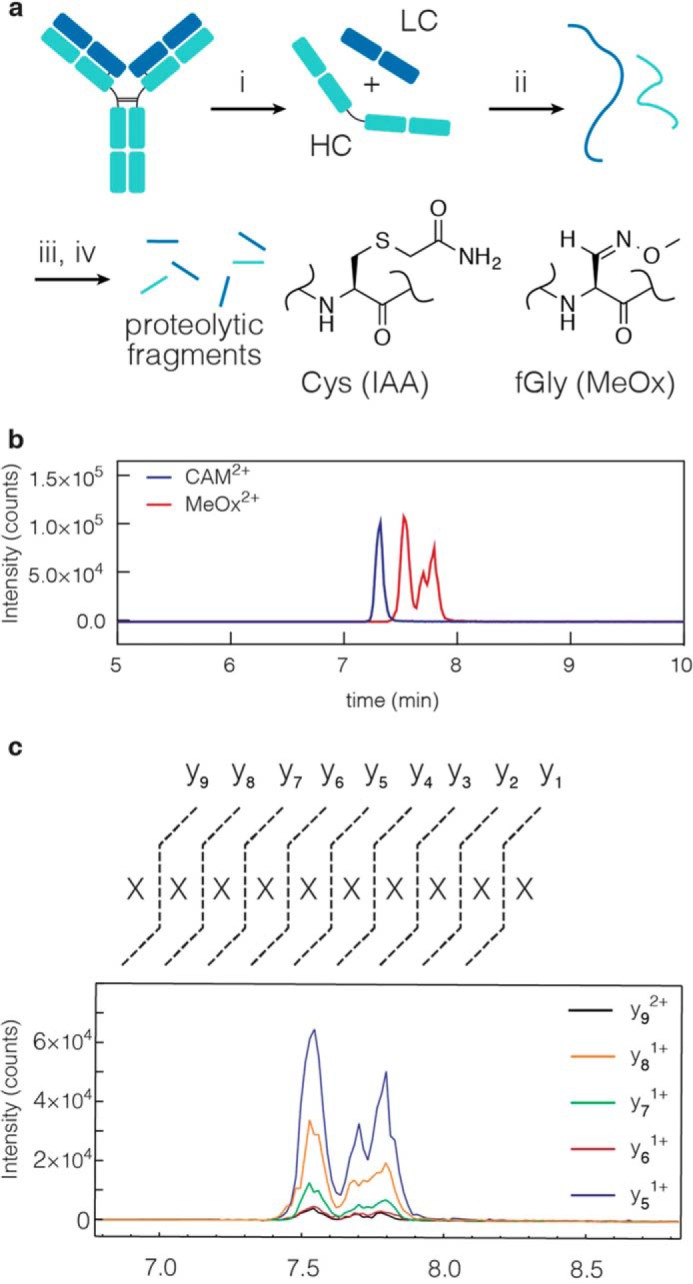
**LC-MRM/MS characterization of Cys- and fGly-containing proteins.** *a*, monoclonal antibody is disassembled and digested, and the Cys and fGly functional groups are trapped as the CAM and MeOx species, respectively, for characterization, through the following steps: (i) DTT, 37 °C, 15 min; (ii) HCl, then NH_4_HCO_3_; (iii) trypsin, iodoacetamide, pH 8, 37 °C, 1 h; (iv) methoxylamine, pH 5.5, 16 h. *b*, each aldehyde tag location is characterized by a unique tryptic peptide containing the installed tag sequence. Here, a 9-mer peptide is generated by trypsin. Targeted MRM/MS was used to quantify the peptides modified as CAM or MeOx. The five most intense precursor/product ion transitions were chosen for peak area integration. *c*, ion chromatogram of the CAM- and MeOx-containing peptides. The MeOx-containing peptide separates into the diastereomers present as a result of oxime formation.

**FIGURE 13.**
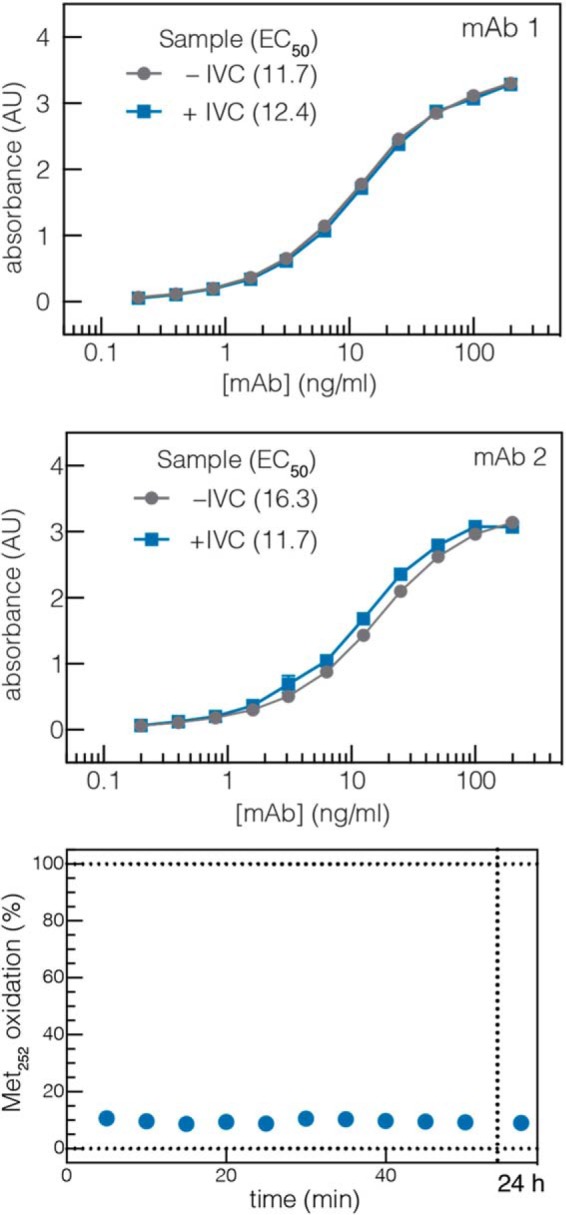
**Biophysical properties of mAbs subjected to *in vitro* conversion reaction conditions.** *Top* and *center*, ELISA measuring antibody/antigen affinity for two IgG1 mAbs. *Bottom*, measurement of the proportion of oxidized methionine in the C_H_3 region (FcRn binding domain) of an IgG1 scaffold is unchanged over the course of the reaction.

After determining this dual role of reducing agent during catalysis, we were able to finalize reaction conditions that smoothly translated to protein substrates. As we have previously described, the aldehyde tag can be introduced at the N or C terminus or at any solvent accessible internal sequence (11). Here, Cys was converted to fGly in high yield by *Hs*-cFGE in three independent regions of a mAb (Fig. 11*a*) and across a wide range of reaction scales (from 0.8 to 200 mg) on three independent mAbs (Fig. 11, *b* and *c*). In each case, the *in vitro* conversion yield was >90%, as measured by LC-MRM/MS (Fig. 12). Furthermore, the pH 9 reaction conditions did not affect antigen binding affinity (Fig. 13, *a* and *b*) or oxidation at Met^252^ (Fig. 13*c*), a residue that is important for FcRn binding and mAb circulation *in vivo* (43, 44).

## Discussion

Formylglycine-generating enzyme was produced by recombinant expression for the generation of aldehyde-tagged proteins *in vitro*. We successfully developed a discontinuous HPLC assay to measure the kinetic parameters of both FGEs. In contrast to previous work, these results indicate that the N-terminal extension of *Hs*-FGE is not required for catalytic activity *in vitro*. Surprisingly, when purified from insect cell culture, the human enzyme was much more active than the bacterial form of FGE from *S. coelicolor* that was produced in *E. coli*.

We also designed experiments to explore the mechanism of FGE turnover. The existing mechanistic hypotheses focus on the redox state of the FGE active site cysteine residues as revealed by crystallography. When *Hs*-cFGE was isolated previously, Cys^336^ (Cys^272^ in *Sc*-FGE) and Cys^341^ (Cys^277^) were present in the enzyme in a mixture of reduced and oxidized forms (8). The oxidation state of Cys^336^ and Cys^341^ can be manipulated using chemical reagents in solution, either to form the Cys^336^-Cys^341^ disulfide, or with H_2_O_2_ over a longer period of time, to form a sulfonic acid at both positions. In addition, a C336S mutation enables the “trapping” of C_sub_ as an intermolecular disulfide with Cys^341^ (6). This convincing set of evidence prompted the hypothesis that FGE uses a thiol-disulfide interchange to anchor C_sub_ and activate Cys^336^. This residue would then perform the activation of molecular oxygen to form a higher oxidation state Cys residue (either sulfenic acid or cysteine peroxide) and subsequently oxidize the substrate.

In this work, we demonstrated that the presence of a disulfide of the active site cysteines does not correlate with enzyme activity. Instead, we made the fortuitous discovery that FGE incorporates copper as a cofactor for catalysis. Our results indicate that recombinant expression of FGE most often results in the apoenzyme, which can be reconstituted as the holoenzyme by the addition of copper(II) sulfate at pH 7. This reconstitution was ineffective with other metal ions commonly found in enzymes, including Mn^2+^, Fe^2+^, and Zn^2+^. Interestingly, the only previous characterization of FGE metal content identified 0.09 copper/human FGE as produced in the mammalian HT1080 cell line (7). We observed that the catalytic rates of both human and bacterial FGE were significantly increased upon activation with stoichiometric amounts of Cu^2+^, and that turnover was inhibited by the addition of cyanide to FGE reaction mixtures. Previous reports have shown that the inhibition of aerobic copper oxidases by CN^−^ results from ligation of CN^−^ at the metal center (in the reduced state of Cu^1+^), which prevents oxygen binding and substrate activation (38). Furthermore, FGE activity was extinguished completely after treating the enzyme with a combination of a chelator and a reductant, reinforcing the highly specific and tight binding interaction between FGE and Cu^2+^.

Taken together, we hypothesize that FGE resides in the well studied class of copper oxidases that perform 2 H^+^/2 *e*− oxidation, activate molecular oxygen, and require exogenous reductants to complete a catalytic cycle. This also helps explain how FGEs that reside in subcellular compartments with varying redox environments can still perform catalysis, turnover depends upon copper and oxygen, and not the presence of an intact disulfide.

More specifically, FGE most closely parallels the structure and reactivity of the Cu_M_ site in enzymes like peptidylglycine α-hydroxylating monooxygenase, which hydroxylates a C terminally extended peptide (42). In peptidylglycine α-hydroxylating monooxygenase (and other related enzymes), the Cu_M_ is the location of oxygen activation, consumption of a molar eq of reducing agent, and 2 H^+^/2 *e*^−^ substrate oxidation.

Despite these similarities to existing mononuclear copper oxidases, FGE remains unique. In Cu_M_, His and Met bind the copper atom, but not a Cys (42). The mononuclear copper-binding motif in FGE is likely a combination of the ligands that are highly conserved, and those whose mutation results in decreased or eliminated activity. Specifically, the residues whose mutations are known to change FGE activity are Cys^336^, Cys^341^, Ser^333^, His^337^, and Tyr^340^.

With respect to cysteine, other enzymes in which isolated copper centers are ligated by thiols (*e.g.* blue copper proteins), the copper center typically performs 1-electron redox chemistry, and not oxygen activation or substrate oxidation (45). In addition, the ligand-to-metal charge transfer of the Cys results in a strong absorption band in the visible spectrum of the enzyme. However, reconstituted FGE holoenzyme does not have any absorption bands above 280 nm, and so it is unlikely to contain a type I copper center. Despite this, mutation of Cys^336^ and Cys^341^ have a significant effect on FGE activity. Their role in the copper active site is unclear.

What we now believe is the FGE apoenzyme is well characterized by x-ray crystallography (6, 8, 9, 20, 21, 46). With respect to other FGE residues, His^337^ seems to be a likely candidate for copper ligation because of the prevalence of His in copper active sites, its proximity to the active site, and the decrease in activity that accompanies its removal. In addition, the highly conserved Tyr^340^ may also participate in the FGE active site, as it does in other oxygen-activating copper oxygenases (23). In galactose oxidase, tyrosine is both a copper ligand (Tyr^495^) and also a redox-active participant (cross-linked Cys-Tyr^272^) in catalysis (47). In the family of copper amine oxidases, the active site contains a TPQ cofactor (a post-translationally modified form of Tyr) that participates in catalysis (48).

**FIGURE 14.**
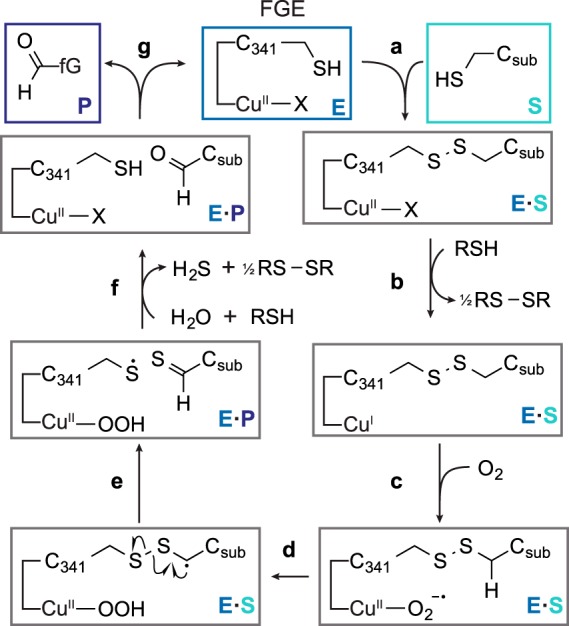
**The proposed catalytic mechanism for FGE that accounts for the copper cofactor.** *a*, substrate binds enzyme and is covalently attached through Cys^341^. This covalent bond formation may be catalyzed by Cu^2+^, or it could result from thiol-disulfide exchange between an existing Cys^341^-reductant disulfide and C_sub_. *b*, reduction of Cu^2+^ to Cu^+^ would enable binding of molecular oxygen (*c*) as the Cu^2+^ superoxo intermediate, as shown for other oxygen-activating copper oxidases (23). *d*, oxidation of substrate by the Cu(II)–O_2_^−^ through proton-coupled electron transfer would generate a disulfide radical, which would rapidly collapse (*e*) to thioaldehyde and thiyl radical. *f*, second 1 H^+^/1 *e*^−^ reduction and hydrolysis would regenerate Cys^341^. The way in which this reduction occurs could proceed through a number of pathways, including, but not limited to: direct thiyl radical reduction and hydrolysis of copper-peroxide to release H_2_O_2_; or oxo transfer from Cu(II)-OOH to the radical to generate a sulfenic acid, followed by copper-oxyl reduction and sulfenic acid hydrolysis. *g*, after product dissociation, the catalytic cycle is complete.

From the data described above, we propose a mechanism by which FGE converts Cys to fGly that accounts for the copper cofactor. As demonstrated above, the formation of side products confirms that *E*·S involves a covalent Cys^341^-C_sub_ (*H. sapiens* numbering) disulfide bridge (Fig. 14*a*). Reducing agent would serve to generate the Cu(I) state of the active site (Fig. 14*b*), which is a well studied (23) intermediate for binding of molecular oxygen (Fig. 14*c*) as the cupric superoxo Cu(II)–O_2_^−^ that is poised for substrate oxidation. A C-H bond on the C_sub_ side chain could then react by proton-coupled electron transfer (49, 50) with the Cu(II)–O_2_^−^ (Fig. 14*d*). After oxidation, the radical on the C_sub_ side chain would almost certainly collapse to the thioaldehyde and a Cys^341^ thiyl radical (Fig. 14*e*). A second equivalent of 1 H^+^/1 *e*^−^ would regenerate the thiol on Cys^341^, and as previously proposed, the thioaldehyde would hydrolyze to the product fGly (Fig. 14, *f* and *g*) and H_2_S. Formally, the oxidation reaction could be complete simply by displacing H_2_O_2_ as a reaction product, but the cupric peroxide Cu(II)–OOH might also undergo further reduction to generate only H_2_O as a byproduct. We would predict that the resting state ligand on copper is a hydroxide, although we cannot rule out direct involvement of Cys^341^ through an alternative mechanism. These scenarios will serve as a guide, but it is clear that developing a comprehensive catalytic cycle for FGE will require additional structural and paramagnetic spectroscopic studies of the enzyme.

With the mechanistic knowledge described above, we optimized the reaction conditions for efficient production of fGly without disrupting existing intramolecular disulfides in the substrate protein. Interestingly, we found that FGE was able to catalyze the formation of disulfides at the substrate Cys when monothiol reducing agents were present in solution. Furthermore, when dithiol reagents such as DTT are used, the rate of product formation decreased, suggesting that any intermolecular disulfide is quickly abolished by DTT cyclization. This indicates that FGE binds C_sub_ in a disulfide, and that when *k*_cat_ is slower than the rate of reaction with reducing agent in solution, C_sub_ can be released as a disulfide. These data confirm the existing proposal that substrate is anchored during turnover, and optimal turnover requires balancing the type and amount of reductant in solution with product formation.

We have further demonstrated that FGE can be produced in good yield and used as an excellent biocatalyst for the production of aldehyde-tagged proteins *in vitro*. Reactions with FGE can scale across at least 3 orders of magnitude in mass with no decrease in yield, and proceed without perturbing existing disulfides or modifying residues that are important for mAb performance *in vivo*.

## Footnotes

2 The abbreviations used are:

FGE: formylglycine-generating enzyme
MRM: multiple reaction monitoring
TCEP: tris(carboxyethyl)phosphine
CAM: carboxyamidomethyl
MeOx: methyl oxime
NTE: N-terminal extension
fGly: formylglycine
ER: endoplasmic reticulum
*Sc*-FGE: *S. coelicolor* FGE
*Hs*-FGE: *H. sapiens* FGE
βME: β-mercaptoethanol
C_sub_: substrate cysteine
C_sub_-βME: substrate Cys/mercaptoethanol disulfide
C_sub_-C_sub_: substrate Cys disulfide dimer
mAb: monoclonal antibody
ICP-MS: inductively-coupled plasmon mass spectrometry
Hs-cFGE: truncated *H. sapiens* FGE
TEAM: triethanolamine
PBS-T: PBS-Tween
DHAA: l-dehydroascorbic acid.
